# MicroRNA: A Dynamic Player from Signalling to Abiotic Tolerance in Plants

**DOI:** 10.3390/ijms241411364

**Published:** 2023-07-12

**Authors:** Ziming Ma, Lanjuan Hu

**Affiliations:** 1 Jilin Provincial Engineering Laboratory of Plant Genetic Improvement, College of Plant Science, Jilin University, Changchun 130062, China; 2 Plant Genetics, TUM School of Life Sciences, Technical University of Munich (TUM), Emil Ramann Str. 4, 85354 Freising, Germany; 3 Max-Planck-Institute of Molecular Plant Physiology, Am Muehlenberg 1, 14476 Potsdam-Golm, Germany

**Keywords:** microRNA, target gene, plant growth and development, signal, abiotic tolerance

## Abstract

MicroRNAs (miRNAs) are a class of non-coding single-stranded RNA molecules composed of approximately 20–24 nucleotides in plants. They play an important regulatory role in plant growth and development and as a signal in abiotic tolerance. Some abiotic stresses include drought, salt, cold, high temperature, heavy metals and nutritional elements. miRNAs affect gene expression by manipulating the cleavage, translational expression or DNA methylation of target messenger RNAs (mRNAs). This review describes the current progress in the field considering two aspects: (i) the way miRNAs are produced and regulated and (ii) the way miRNA/target genes are used in plant responses to various abiotic stresses. Studying the molecular mechanism of action of miRNAs’ downstream target genes could optimize the genetic manipulation of crop growth and development conditions to provide a more theoretically optimized basis for improving crop production. MicroRNA is a novel signalling mechanism in interplant communication relating to abiotic tolerance.

## 1. Introduction

Today’s population is growing exponentially, and new arable land is needed to grow food and increase food production in order to feed future generations. As the population grows, there is also an increased shortage of energy and a need to produce alcohol as a new source of energy through the fermentation of crops. However, many environmental conditions have changed, exposing plants to a wide range of abiotic stresses and greatly affecting plant growth and development. Abiotic stresses, such as drought and salinity, are known to significantly affect plant survival, growth and development, thereby reducing plant quality and biomass production. The effects of abiotic stresses may also be reflected at different sub-biotic levels, including biochemical, physiological, cellular, molecular and even biological levels. Studies have shown that many genes can enhance plant tolerance in response to abiotic stresses when overexpressed in plants [1,2,3]. However, many questions remain unanswered, as follows: How do these genes regulate plant tolerance? Which gene networks do plants use to cope with different abiotic stresses? Is the expression of these resistance genes regulated by other genes? A recently discovered small regulatory RNA molecule, called microRNA (miRNA), may be the answer to these questions.

miRNAs, a class of plant non-coding single-stranded RNA molecules approximately 20–24 nucleotides in length encoded by endogenous genes, have a variety of important regulatory roles in cells, participating in the regulation of plant growth and development, stress responses and hormone signalling through the negative regulation of plant gene expression, and post-transcriptional regulation of gene expression in plants [4]. miRNAs can complementarily bind to the 3’UTR (untranslated region) region of the target messenger RNA (mRNA), thus achieving negative regulation of gene expression. Several miRNAs can also regulate the same gene and be regulated by a combination of several miRNAs. It has been shown that miRNAs are not only conserved in the gene location, but also exhibit a high degree of sequence homology. This high degree of conservation is closely related to their functional importance and may suggest that homologous miRNAs have similar functions in different species [5,6,7]. Since the discovery of miRNAs in 1993, an increasing number of researchers have become interested in such non-coding RNAs [6].

Plants respond to stress, such as cold and drought, by activating internal stress defence mechanisms that stimulate physiological responses. For example, overexpression of several stress-responsive genes, including *OsGATA16* and *OsWRKY55*, leads to physiological changes that enhance cold tolerance and drought resistance [1,2]. Abscisic acid (ABA) has been shown to play a key role in the regulation of drought tolerance and seed germination in plants. ABA accumulates in response to abiotic stresses and promotes *miR159* expression. MiR159 is an ancient and conserved plant miRNA that plays multiple roles in plant development and drought response. Jiang et al. showed that a loss-of-function mutation in the *ABI5* gene suppressed the hypersensitivity of *miR159* to ABA and that the insensitivity of *myb33* seeds to ABA treatment was *ABI5*-dependent. *ABI5* functions downstream of *MYB33* and miR159 in response to ABA [8]. Not only can miRNAs improve plant tolerance to abiotic stresses by regulating downstream genes; recent studies have shown that miRNA is a novel signalling mechanism in interplant communication relating to abiotic tolerance. Betti et al. tested the hypothesis that miRNA can transfer from one plant to another by using two mobile miRNAs and their targets (miR399/*PHO2* and miR156/*SPL*). This ultimately suggests that miRNA can be transported between plants, that it may be involved in interplant communication and that miRNA can act as a signal to regulate phosphorus nutrient stress in plant [9].

In this review, we describe the mechanisms of miRNA production and its actions, as well as miRNAs that have now been identified as being able to respond to different types of abiotic environmental stresses and miRNAs acting as a novel signalling mechanism for interplant communication involving abiotic stresses. In the future, we can focus more research on miRNAs as a network of signalling regulatory genes in response to adversity to improve plant tolerance to abiotic stresses from a signalling perspective, with a view to improving crop yields.

## 2. Biosynthesis and Mode of Action of Plant miRNAs

### 2.1. Biogenic Pathways of Plant miRNAs

miRNAs are transcribed by RNA polymerase II, and the initial transcription products are called primary transcription products of miRNAs (pri-miRNAs). Many pri-miRNAs have the same 3’-poly(A) and 5’-cap structures as the transcripts encoding the genes, and some pri-miRNAs also contain intron structures; one of the most important properties is the ability of miRNAs to form hairpin-shaped stem–loop structures. Pri-miRNA is very long, ranging from a few hundred to several thousand bases in length. Pri-miRNA maintains its non-activated state via its cap structure and polyadenylation and coiling of the spatial structure. Pri-miRNA is transcribed and sheared into pre-miRNA containing a stem–loop structure. Pri-miRNAs are composed of a single hairpin structure. The nucleoplasmic transporter protein recognizes the two nucleotides protruding from the 3’ ends of the pre-miRNA hairpin sequence and transports the pre-miRNA from the nucleus to the cytoplasm. After further processing to form miRNA double strands (miRNA/miRNA*), pre-miRNA is processed by a cleavage complex containing Dicer-like I (DCL1), HYPONASTIC LEAVES1 (HYL1) and SERRATE (SE) as core components. DCL1 acts as a shearing agent, cutting pre-miRNA into 21 nt miRNA/miRNA* double-stranded bodies, and HYL1 interacts with DCL1 to facilitate efficient and precise pri-miRNA processing. Another protein, DAWDLE (DDL), was also found to interact with DCL1 to regulate pre-miRNA processing. The double-stranded body is translocated into the cytoplasm, and the pre-miRNA is processed into a mature double-stranded miRNA/miRNA* complex with the help of the HUA ENHANCER 1 (HEN1) protein helper complex. HEN1 encodes a methyltransferase and plays an important role in the methylation of miRNAs during this process. In plants, miRNA shearing and processing is carried out in the nucleus, but most mature miRNAs perform their functions in the cytoplasm, and plant HST (HASTY) proteins are able to transport miRNA/miRNA* from the nucleus to the cytoplasm to perform their functions. HASTY protein has been thought to function as a transporter protein in this process [10,11,12]. However, it is unclear whether miRNAs are transported to the cytoplasm prior to RNA-induced silencing complex (RISC) formation in this synthetic mechanism. In earlier models, miRNA/miRNA* double-stranded bodies were transported to the cytoplasm via HASTY protein and then loaded onto ARGONAUTE1 (AGO1). However, an alternative model has recently been proposed in which loading of AGO1 occurs in the nucleus [13].

### 2.2. Mode of Action of Plant miRNAs

Plant miRNAs regulate target genes at the post-transcriptional level through two mechanisms: degradation of target gene mRNA (more common) and translational repression of target genes (less common).

**Figure 1:**
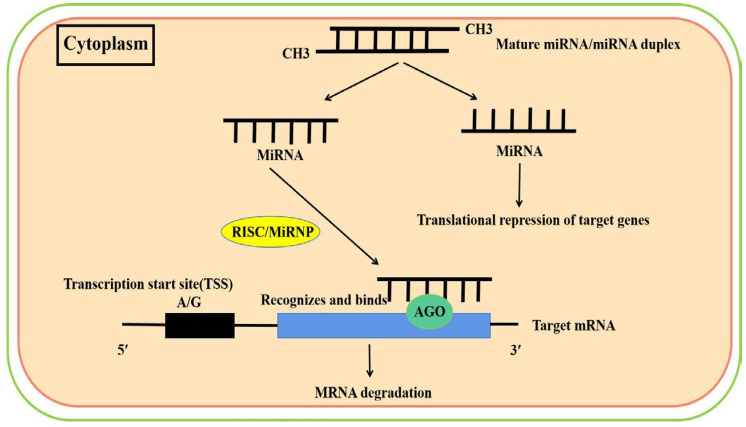
The mode of action of plant miRNAs: after the plant miRNA recognizes and binds to the target mRNA, the AGO will shear the target mRNA at the 10th and 11th nucleotides of the miRNA binding site. When plant miRNAs bind to target mRNAs, the AGO will shear the target mRNA of the miRNA binding site, thereby degrading the target mRNA. Another mechanism of plant miRNAs action is achieved through translational repression of its target genes.

In the degradation of target gene mRNA, mature miRNAs need to enter an RISC in order to function. This complex is also known as the miRNA–ribonucleoprotein complex (miRNP), which contains both mature miRNAs and proteins. The ARGONAUTE (AGO) protein is the most important protein in this RISC complex. Since most plant miRNAs are derived from the reverse copy of the target genes, the bases of the miRNAs are complementary to those of the target mRNAs. After the plant miRNA recognizes and binds to the target mRNA, the AGO will shear the target mRNA at the 10th and 11th nucleotides of the miRNA binding site. Ten AGOs have been identified in *Arabidopsis*; AGO1 has four structural domains, PAZ, Mid, PIWI and the N-terminal domain, respectively. Numerous studies have demonstrated that complete complementary pairing of bases near the plant miRNA shear site with the target mRNA is necessary for AGO1 to achieve its shear function (Figure 1). Franco et al. found that the transcript of *IPS1* (*Induced by phosphate starvation 1*, a non-coding gene) was recognized and bound by miR399, but could not pair with miR399 at the miR399 splice site, resulting in the inability of AGO1 to splice it. Based on this principle, researchers have designed a variety of transcripts that can bind to target miRNAs but cannot be cut by AGO1 (Figure 1) [14,15,16].

Another mode of action of plant miRNAs is achieved through translational repression of target genes (Figure 1). Aukerman et al. found that overexpression of *miR172* did not reduce the expression abundance of target mRNAs, but the corresponding levels of proteins encoded by target mRNAs were significantly reduced [17]. Therefore, they proposed that plant miRNAs can also repress the translation of target genes, and the same miRNA may even regulate target genes in both a shearing and translationally repressed manner. This is one of the reasons why the expression of plant miRNAs and their target genes is not fully complementary [18,19].

## 3. Overall Role of Plant miRNAs in Response to Drought Stress

Water is a vital resource for the survival of all life and has played an important role in the evolution of life. The most abundant substance in plant cells is water, which is an essential component of the plant body. With sufficient water, the stalks and branches of plants can stand up and stretch in the air, and the flowers can bloom better and facilitate the completion of pollination. Water is also one of the raw materials necessary for photosynthesis in green plants, and if there is a lack of water, the plant’s photosynthesis process will be weakened. Leaves will wilt, and in severe cases, it can lead to the death of the plant [20,21,22]. MiR156 was one of the first miRNAs identified in plants, and numerous studies have linked miR156 to drought stress. Anthocyanins act as a secondary metabolite by scavenging reactive oxygen species (ROS) to protect plants from stress. MiR156/SPL is present in *Arabidopsis*, rice, alfalfa and poplar, regulating anthocyanin accumulation levels in response to plant drought stress. Plant drought stress triggers this mechanism to regulate the level of anthocyanin accumulation [23,24]. López-Galiano et al. showed that drought conditions lead to the downregulation of miR159 and upregulation of its target gene transcription factor *MYB33* in tomato [25]. Reyeset et al. found that during *Arabidopsis* seed germination, ABA induces the accumulation of miR159 in an ABI3-dependent manner, and miR159 mediates the cleavage of *MYB101* and *MYB33* transcripts in vitro and in vivo [26]. Zhang et al. showed that fine localization and functional analysis identified the candidate gene *ZmLRT* of *qLRT5-1* as expressing the major transcript of miR166a, and that the knockdown of *ZmLRT* lines enhanced drought tolerance in maize seedlings [27]. Stomata play a central role in the exchange of gases between plants and their environment, and stomata opening and closing are influenced by environmental signals, as well as being regulated by endogenous hormones, which in turn affects the plant’s response and tolerance to drought stress. ABA is the most critical hormone in drought stress, regulating water loss and stomatal opening and closing [28,29,30,31]. MiR393 positively regulates stomatal density and negatively regulates guard cell length, while overexpressing lines have the opposite phenotype to the deletion mutant, possibly due to miR393 regulating the expression of *ARF5* and two stomatal-development-related genes, *EPF1* and *SPCH*. The *miR393*-overexpressing line is more sensitive to drought treatment, accumulating more malondialdehyde (MDA) and hydrogen peroxide (H_2_O_2_) compared to the wild type, and also inhibiting the accumulation of ABA in leaves. These results also demonstrate that miR393 responds to plant drought stress by interacting with ABA and regulating stomatal density [32]. Zhao et al. found that the overexpression of *miR393a* enhanced drought stress tolerance associated with stomatal density and epidermal densification. MiR393 regulates the expression of *AUXIN SIGNALLING F-BOX 2 (AsAFB2)* and *TRANSPORTINHIBITOR RESPONSE 1 (AsTIR1)* [33]. To adapt to drought stress, plants require a hormone monocrotaline lactone, and exogenous monocrotaline lactone applied to tomato induces the accumulation of miR156. The overexpression of *miR156* and monocrotaline lactone treatments both result in reduced stomatal conductance and increased ABA sensitivity in plants [34]. MiR398c was able to negatively regulate drought resistance in soybean. The overexpression of *miR398c* reduced the expression of *GmCSD1a/b*, *GmCSD2a/b/c* and *GmCCS* in *Arabidopsis*; impaired the plant’s ability to scavenge active oxygen; and increased relative electrolyte leakage and stomatal opening. This reduced germination and increased water loss from the leaves, and at the same time this reduced survival and led to sensitivity to drought during seed germination and seedling growth [35]. Plants can also improve their drought stress tolerance by changing root conformation and adjusting leaf size and curl. Hang et al. showed that *OsmiR408*-transgenic plants have increased drought tolerance, which may be due to changes in their leaf morphology that facilitate the maintenance of water status, as well as their increased antioxidant capacity to protect against damage from ROS under stress [36]. Wang et al. found that *miR9674a* showed progressive upregulation in response to drought stress treatment. *MiR9674a*-expressing lines exhibited different growth characteristics under drought and salt treatment in tobacco, with significant improvements in plant biomass, leaf area and root length, while its knockout *miR9674a* lines showed significant alleviation in the above growth traits compared to the wild type [37].

## 4. Regulating Mechanism of Plant miRNAs in Response to Salt Stress

Soil salinity affects around 6% of all land and 23% of arable land, causing considerable economic losses through crop stress and reduced yields. Because salinity plays a vital role in plant growth, above a certain limit, excess soluble salts will have a toxic effect on plants. Quinoa can use antioxidants to scavenge excess ROS; it has high uptake and retention of K^+^, Ca^2+^ and Mg^2+^ as charge-balancing ions, increases stomatal density (SD) and decreases stomatal aperture (SA) to maintain photosynthesis (Pn), leading to improved growth under salinity. In addition, the accumulation of excessive salt in plants can also affect the levels of endogenous plant signalling molecules such as ABA, ethylene, gibberellin (GA) and nitric oxide (NO). Once these signalling molecules are affected, they can greatly inhibit plant growth and development, ultimately leading to a reduction in yield [38,39].

In recent years, a number of miRNAs have been identified through miRNA studies on plant response to salt stress. The increased abundance of miR399 under salt stress, and therefore the altered expression of target genes’ *PHO2*, resulted in significant changes in the expression levels of two transporter genes, *PHOSPHATE TRANSPORTER1;4 (PHT1;4)* and *PHT1;9*. Salt-stressed *Arabidopsis* enhances PO_4_ transport from the roots to shoot tissues, and these aerial tissues can use these resources to maintain essential biological processes or to generate adaptive responses under salt stress [40]. PpDCL1a encodes an essential dicer protein for miRNA biogenesis and contains an intron miR1047. Precise deletion of the intron containing miR1047 to abrogate PpDCL1a autoregulatory feedback control revealed a hypersensitive response to salt stress and an insensitive response to the phytohormone ABA, as well as the physiological importance of feedback control of miR1047 for the abundance of PpDCL1a transcripts, which controls miRNA expression and its homologous target gene RNAs during salt stress adaptation [41]. The overexpression of *sly-miR398b* inhibited plant growth under salinity conditions in tomato, including that above-ground and root biomass, and led to a shorter plant height. Further analysis showed that overexpression of *sly-miR398b* downregulated the expression of Cu/Zn superoxide dismutase (CSD) [42]. Liu et al. identified two contrasting *Fraxinus velutina* var. *velutina* cutting clones, one of which was salt-tolerant (R7) and the other salt-sensitive (S4), and found that R7 exhibited higher salt tolerance than S4. In R7 leaves, miR164d, miR171b/c, miR396a and miR160g targeting *NAC1*, *SCL22*, *GRF1* and *ARF18*, respectively, were involved in salt tolerance. In R7 roots, miR396a, miR156a/b, miR8175, miR319a/d and miR393a targeting *TGA2.3*, *SBP14*, *GR-RBP*, *TCP2/4* and *TIR1*, respectively, were also involved in salt stress response [43]. Yuan et al. found that *Osa-miR396c*-overexpressing lines exhibited reduced biomass, leaf area and leaf size and shorter internodes compared with the wild type, while the transgenic plants showed increased water retention under high salt stress [44].

## 5. Role of Plant miRNAs in Response to Temperature Stress

### 5.1. miRNA and Low-Temperature Stress in Plants

Temperature is the main environmental factor affecting plant growth and development and the quality of life of the fruit after harvest. Low temperature can inhibit plant growth and is a very important abiotic stressor. A variety of miRNAs are involved in the low-temperature stress response of plants by affecting the IAA or ABA signalling pathway [45]. Wang et al. showed that miR319 targets the *TEOSINTE BRANCHED/CYCLOIDEA/PCF (TCP)* transcription factor genes, which are involved in regulating multiple processes in plant growth and development by controlling cell proliferation. *MiR319* expression is downregulated by low-temperature induction, while its target genes *OsPCF6* and *OsTCP21* are reversed, and the overexpression of *miR319* enhances lines’ tolerance to low-temperature stress [46]. Overexpression of *miR156* resulted in increased cell viability and growth rate under cold stress in *Arabidopsis*, pine and rice. MiR156 increased plant cold tolerance by targeting *OsSPL3*, which positively regulates the expression of *OsWRKY71*, a negative regulator of the transcription factor genes *OsMYB2* and *OsMYB3R-2* [47]. Dong et al. found that SlNAM3 enhances cold tolerance and Sl-miR164a/b-5p plays a negative role in cold tolerance by repressing the expression upstream of *SlNAM3*. The SlmiR164a-SlNAM3 module induces ethylene synthesis by directly regulating the expression of *SlACS1A*, *SlACS1B*, *SlACO1* and *SlACO4*, thereby increasing cold tolerance in tomato [48]. The APETALA2/ethylene response factor (ERF) transcription factor *OsERF096* was identified as a target gene of miR1320 that negatively regulates cold stress tolerance. The overexpression of *miR1320* increases lines’ cold tolerance, while the knockdown of *miR1320* decreases lines’ cold tolerance. The miR1320-OsERF096 module regulates cold tolerance by inhibiting the jasmonate-mediated cold signalling pathway [49].

### 5.2. miRNA and High-Temperature Stress in Plants

The response of plants to temperature stress is a complex process involving a variety of metabolic and biochemical processes. While low temperatures affect plant growth and development, high temperatures also negatively affect processes such as growth, development and reproduction [50,51,52,53,54,55]. Wang et al. found that SRL10, a double-stranded RNA-binding protein, regulates leaf morphology and heat tolerance in rice by altering miRNA biogenesis. The *srl10* mutant has a semi-curled leaf phenotype and increased heat sensitivity. SRL10 interacts directly with catalase isozyme B (CATB) to enhance hydrogen peroxide (H_2_O_2_) scavenging, thereby promoting heat tolerance [56]. Li et al. showed that overexpression of *miR9748* increased the high-temperature tolerance of *Arabidopsis thaliana*. Transcriptome analysis suggests that miR9748 may mediate high-temperature tolerance through the phytohormone signalling pathway. The target gene of miR9748 is *CsNPF4.4*, which negatively regulates high-temperature stress tolerance by repressing the jasmonate signalling pathway [57]. Ahmed et al. found that novel and conserved heat-responsive miRNAs were identified in Chinese cabbage using a high-throughput sequencing approach involving a heat stress treatment at 38 °C. This analysis identified 41 conserved miRNAs from 19 families, with miRNA156, miRNA159, miRNA168, miRNA171 and miRNA1885 having the most abundant molecules [58].

## 6. miRNAs Involved in Plant Response to Heavy Metals

Excessive accumulation of heavy metals can cause toxicity in plants, affecting plant growth and development. Crops poisoned by heavy metals induce cell damage in humans and animals through the food chain, leading to disease. Metal elements include essential and non-essential elements. Essential metals such as zinc, manganese and copper are required for many physiological processes in living organisms, while non-essential metals include cadmium, lead and mercury [59,60,61,62,63,64,65,66,67,68,69]. Zhang et al. found that *miR156*-overexpressing lines accumulated significantly less Cd in their branches and showed enhanced tolerance to Cd stress in plants. The reason for this is that miR156 positively regulates Cd stress tolerance by regulating ROS levels and Cd uptake/transport gene expression [70]. Lines overexpressing *miR408* showed severe susceptibility to low sulphur (LS), arsenite As(III) and LS + As(III) stresses due to their altered state, and *miR408* knockout lines showed tolerance due to the regulated expression of genes involved in the sulphur reduction pathway, affecting the accumulation of sulphate and glutathione [71]. Nie et al. showed *miR167a*, *novel_miR15*, *novel_miR22* and their targets may be involved in Cr transport and chelation. In addition, *miR156a*, *miR164*, *miR396d* and *novel_miR155* were identified as being involved in the detoxification of plant Cr [72]. Zhou et al. found, by comparing miRNAs and transcriptome analysis, a total of 3 known and 19 new differentially expressed *microRNAs* (DEMs) and 1561 differentially expressed genes (DEGs), which were identified following Cd treatment, because miRNAs play an important role in Cd-stressed wheat by regulating targets such as *TaHMA2;1* [73]. *MiR393*-overexpressing lines exhibited severe inhibition of root elongation by aluminium ions. In addition, the overexpression of *miR393* attenuated the effect of exogenous growth hormone on aluminium-induced root growth inhibition and downregulated the expression of growth-hormone-responsive genes under aluminium stress [74].

## 7. Molecular Mechanisms of Plant miRNAs Associated with Nutritional Element Stress

Among macronutrients, the most crucial nutrients are nitrogen (N), phosphorus (P) and potassium (K), which play important roles in the growth and development of plants [75,76,77,78]. Nitrogen is a major component of many important compounds in plants, participating in a range of biochemical reactions and playing a key role in crop biomass accumulation and yield enhancement [79]. Phosphorus is involved in photosynthesis, respiration, energy storage and transfer, cell division, cell enlargement and a number of other processes in plants [80]. Potassium is involved in osmoregulation, material transport and other processes, and can improve stress tolerance in plants [81]. Lyzenga et al. found that nutrient deficiencies cause plants to exhibit a reduced dry weight of tissues in the above-and below-ground parts and reduced root length, root surface area, root volume, root vigour and root respiration. Therefore, a deficiency of nutritional elements greatly affects plant growth and causes plant death in severe cases [82].

Nitrogen: Many previous studies have shown miRNA production is induced in response to nitrogen. MiR167 is able to limit root growth, because it controls the response of adventitious plants to N and even controls N-metabolizing enzymes produced downstream of nitrification and uptake [83]. MiR393 is activated by N signalling transmitted during nitrification and uptake. Nitrate has no effect on primary root development in *miR393*- or *afb3-1*-mutant-overexpressing lines, but it controls horizontal root development in response to nitrate treatment [84,85].

Phosphorus: miR399 is an important component of the phosphorus starvation signalling pathway. The function of miR399 in phosphorus starvation signalling was first elucidated in *Arabidopsis*. M*iR399* expression was increased under phosphorus starvation conditions, increasing the uptake and translocation of inorganic phosphorus in response to phosphorus deficiency [86]. Hu et al. showed that alongside the upregulation of genes in response to phosphorus starvation, many genes involved in iron, potassium, sodium and calcium uptake were also significantly upregulated in *miR399*-overexpressing lines, with increased concentrations of iron, potassium, sodium and calcium. In addition, the *Ospho2* mutant also resulted in increased concentrations of these nutrients as well as the upregulation of related genes. This demonstrates that miR399 influences plant responses to nutrient stress by regulating *OsPHO2* expression [87].

Potassium: Researchers have demonstrated that miRNA expression in cotton and wheat is altered by low dietary potassium utilization. K-deficiency treatment resulted in altered expression of 16 of the 20 miRNAs. In response to K deficiency, wheat increases root growth and nutrient uptake through molecular mechanisms. In peanut plants, root development is influenced by miRNAs, which play a key role in K-deficiency conditions. *MiR156* and *miR390*, together with *miR160*, *miR164* and *miR393*, are proposed to be upregulated in response to potassium deficiency [88,89]. Under low K stress in barley, many miRNAs appear to be differentially expressed, including *Hvu-miR160a*, *Hvu-miR169h* and *Hvu-miR396c*. Due to the induction of miR319 under low K, it is able to repress the expression of the growth response factor gene *HvGRF* and thus promote *Hvu-miR396* transcription in barley [90]. The dormancy-associated MADS-box (OsMADS23) target gene is significantly upregulated in response to potassium deficiency, while *Osa-miR444a* clearly regulates N and P accumulation [91].

Aside from nitrogen, phosphorus and potassium, there are other elements in plants that play key roles in plant growth, such as magnesium (Mg), iron (Fe), sulphate (S), manganese (Mn), copper (Cu) and boron (B). Mg is one of the main components of chlorophyll and promotes the activation of phosphatase and glucose convertase, facilitating the conversion of monosaccharides. Fe is an essential element for chlorophyll formation and is directly or indirectly involved in the formation of chloroplast proteins. S is a protein, amino acid, vitamin and enzyme component that promotes redox and growth regulation and is involved in chlorophyll formation and sugar metabolism. Cu is a core element in the activation groups of various oxidative enzymes in crops and plays an important role in catalysing redox reactions in the crop. Mn is an activator of enzymes and a component of chloroplasts. B is involved in water, sugar and nitrogen metabolism and cell membrane pectin formation. It is also involved in promoting the differentiation of meristematic tissues, the development of flowering organs and seed formation [92,93,94,95,96]. During sulphate limitation, *miR395* expression is significantly upregulated. MiR395 targets two genes capable of participating in the sulphate metabolism pathway, ATP sulfatase (encoded by the APS genes) and sulphate transporter protein 2;1 (*SULTR2;1*, also known as *AST68*) [97]. Valdés et al. found a novel common bean stress response miRNA for manganese toxicity [98]. Kayihan et al. showed the expression level of miRNAs for transcription factors related to jasmonate and ethylene metabolism was significantly increased under moderate B toxicity but not severe B toxicity, with the most significant regulation observed in *Arabidopsis* for *miR172* and *miR319* [99]. Ozhuner et al. identified a total of 31 known miRNAs and 3 new miRNAs in barley; 25 of these were found to be responsive to boron treatment [100]. Thus, miRNAs may regulate the expression of downstream genes to resist stress in plants.

In summary, most research on transgenic technology has focused on the effects of abiotic stress conditions on seed germination and seedling growth. A few studies have focused on the period of crop maturity. Therefore, all of the above transgenic research needs to be applied to crops with a focus on the overexpression of individual miRNAs during crop maturation to provide a basis for the use of novel miRNA-based biotechnologies to improve crop tolerance to various environmental stresses during maturation.

## 8. MicroRNA: A Novel Signalling Mechanism in Interplant Communication

**Figure 2:**
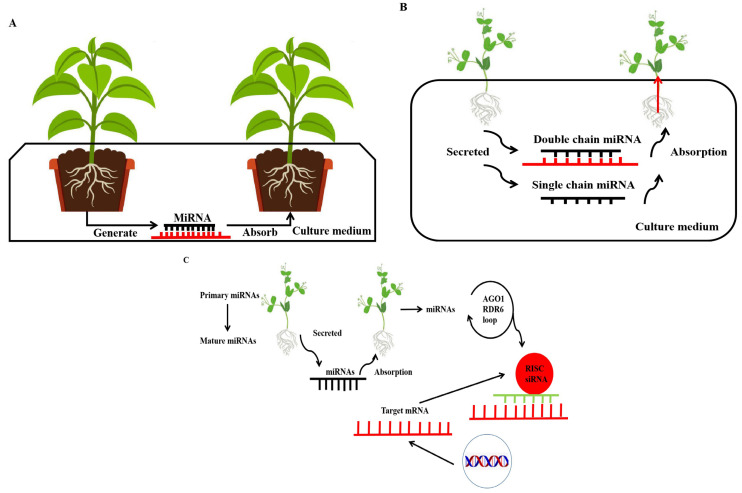
miRNAs act as a novel signalling mechanism in interplant communication. (**A**) When plants are grown in shared growth media, miRNAs are secreted into the media and affect the phenotype of nearby recipient plants. (**B**) miRNAs are secreted as double-stranded or single-stranded mature miRNAs, taken up by the roots of the receiving plant and transferred into the plant via the xylematic route. (**C**) miRNAs are produced in donor plants, where they are processed from primary miRNAs to mature miRNAs and secreted into the external medium according to an unknown mechanism. Root cells of nearby plants can take up these exogenous miRNAs, which are amplified by a loop-induced signal requiring ARGONAUTE1 (AGO1) and RNA-dependent RNA polymerase 6 (RDR6) to produce secondary small interfering RNAs (siRNAs), thereby silencing the target gene in the recipient plant (mRNA, messenger RNA; RISC, RNA-induced silencing complex).

Plants can communicate inter- and intra-specifically by transmitting signals in the form of root secretions and volatiles. Signalling and sensing from neighbouring plants allows plants to gather information about plant parasite hosts and symbiotic partners. The ability for cross-species miRNA trafficking to occur between a parasitic plant and its host plant has been demonstrated by Shahid et al., who showed that the parasitic plant *Cuscuta* uses capillaries to obtain water and nutrients from the host plant. In *Cuscuta campetris*, a large number of miRNAs are induced in the haustorium when it parasitizes *Arabidopsis* and tobacco. These miRNAs can hijack the silencing machinery of host plants, thereby inducing the production of secondary siRNAs and the subsequent degradation of host mRNAs [101]. Betti et al. tested the hypothesis that miRNAs are translocated from one plant to another using two mobile miRNAs and their targets (miR399/*PHO2* and miR156/*SPL*) [9]. They used plant extracts obtained from miRNA-overexpressing (*miR399* or *miR156*) plants to feed *Arabidopsis* seedlings. These extracts were enriched with a specific miRNA, thus allowing the response to be tested in plants with basal expression of the miRNA under study. When treated with extracts containing exogenous miRNAs, wild-type *Arabidopsis* seedlings exhibited downregulation of the miR399 target. In a liquid medium of *miR156*-overexpressing and wild-type lines, the authors obtained the same results, with miR156 being detected in the hydroponic medium, and the expression of its targets, *SPL3* and *SPL9*, was reduced in the wild type. This suggests that plants can take up miRNAs from the medium and thus downregulate their target genes. These exogenous miRNAs can be either extracted from the plant and used as an RNA mixture or chemically synthesized. This ultimately suggests that miRNAs can be transported between plants and may be involved in interplant communication [102,103] (Figure 2). Due to the ability of miRNA to transfer between plants, it is possible to overexpress the miRNA gene that can regulate plant abiotic stress in one plant, and transfer it to another plant through miRNA in order to improve the abiotic stress resistance of both plants, promote plant growth and development and ultimately increase food production.

## 9. Conclusions and Prospects

Environmental stresses, such as drought, salt, temperature, heavy metals and nutritional element stress, affect the metabolic processes of plants, which in turn regulates the expression of secondary metabolites, the synthesis of which reduces the toxic effects of reactive oxygen groups through signal transduction, redox and other mechanisms to ensure the continued survival of the plant (Table 1). A lot of research has shown that different miRNAs are induced in plants responding to different environmental stresses. miRNAs are important regulators in the gene regulatory network and have various functions in regulating the growth, development, programmed cell death and metabolism of organisms [104]. miRNAs can cause changes in the expression of various genes in plants; therefore, these miRNAs can improve the resistance of plants to abiotic stresses. Over the past two decades, researchers have identified a large number of plant miRNAs in major crops and model plants that act in response to abiotic stresses. Considering the continuous improvement in high-throughput and deep sequencing technologies, we can use these sequencing tools to perform genome-wide miRNA expression analysis under abiotic stresses and efficiently and rapidly identify multiple miRNA targets, including degradome sequencing, which enables the identification of the mechanisms of action of a large number of miRNAs [105]. Currently, most studies are focused on the identification of downstream target genes of miRNAs. Little research has been carried out on upstream regulatory elements, and we should pay attention to how upstream regulatory elements regulate miRNAs in the future. Furthermore, people usually focus on miRNA’s response to abiotic stresses such as drought, salt and so on, while the molecular mechanisms of miRNA in response to chemical reagent stress are rarely reported. In today’s increasingly developed industry, chemical pollutants such as car exhaust, haze and pesticides are extremely harmful for crops. The main components of car exhaust and haze are sulphide; these high concentrations of SO_2_ greatly exceed the levels plants can withstand, so an affected plant will exhibit, in a short period of time, leaf scorch and hampered growth and development, until it has withered and died. Excessive use of pesticides can damage the growing environment, increase a crop’s tolerance burden when sprayed on the crop and lead to a reduction in crop yield. As people are now paying more attention to food quality and safety, we were able to focus on functional studies of miRNAs under chemical reagent stress to provide a valuable reference for addressing food security issues. In a recent study, Betti et al. tested the hypothesis that miRNAs are transferred from one plant to another via two mobile miRNAs and their targets (miR399/PHO2 and miR156/SPL) [9]. Ultimately, it was shown that miRNAs can be transported between plants and may be involved in interplant communication [101,102,103]. Therefore, our future research could also focus on miRNAs as a new signalling mechanism in interplant communication with the realization of abiotic stress. Based on the fact that miRNAs can be transferred between plants as a signal, I believe that miRNA genes capable of regulating abiotic stress in plants can be overexpressed in one plant and transferred to another plant via miRNAs to improve abiotic stress resistance in both plants, promote plant growth and development and ultimately increase food production.

In summary, miRNAs are essential for the regulation of mRNA translation in plants, and research exploring the mechanisms of miRNA downstream target gene action could provide a more theoretical basis for improving food production and security.

## Figures and Tables

**Table 1:** Abiotic-stress-responsive miRNAs: their regulation and target genes in plants.

Abiotic Stress Type	miRNA	Expression	Species	Target Genes	References
Drought	MicroRNA-157	Upregulated	*Arabidopsis thaliana*	SPB transcription factor	[106]
Drought	MicroRNA-159	Upregulated	*Arabidopsis thaliana*	MYB and TCP transcription factors	[107]
Drought	MicroRNA-160	Downregulated	*Arabidopsis thaliana*	ARF10, ARF16, ARF17	[108]
Drought	MicroRNA-166	Upregulated	*Medicago truncatula*	HD-ZIPIII transcription factors	[109,110]
Drought	MicroRNA-167	Upregulated	*Arabidopsis thaliana*	ARF6, ARF8	[106]
Drought	MicroRNA-168	Upregulated	*Arabidopsis thaliana*	ARGONAUTE, MAPK	[106]
Drought	MicroRNA-169	Downregulated	*Arabidopsis thaliana*	NF-YA transcription factor, SIMRP1	[111]
Drought	MicroRNA-171	Upregulated	*Arabidopsis thaliana*	GRAS transcription factor	[106]
Drought	MicroRNA-319	Upregulated	*Arabidopsis thaliana*	TCP family	[112]
Drought	MicroRNA-390	Upregulated	*Vigna unguiculata*	ARF family	[113]
Drought	MicroRNA-393	Upregulated	*Arabidopsis thaliana*	(TIR1, AFB2, AFB3) (ARF5, EPF1, SPCH)	[114,115]
Drought	MicroRNA-396	Upregulated	*Arabidopsis thaliana*	GRL transcription factor	[106]
Drought	MicroRNA-397	Downregulated	*Oryza sativa*	Laccase genes	[116]
Drought	MicroRNA-398	Upregulated	*Medicago truncatula*	Superoxide dismutase	[110]
Drought	MicroRNA-398c	Downregulated	*Soybean*	GmCSD1a/b, GmCSD2a/b/c, GmCCS	[35]
Drought	MicroRNA-408	Upregulated	*Arabidopsis thaliana*	Chemocyanin precursor, kinases	[106]
Drought	MicroRNA-474	Upregulated	*Zea mays*	PDH, PPR	[117]
Drought	MicroRNA-528	Downregulated	*Zea mays*	POD	[117]
Drought	MicroRNA-811	Downregulated	*Catharanthus roseus*	MYB transcription factor	[118]
Drought	MicroRNA-814	Downregulated	*Phaseolus vulgaris*	Hydroxyproline-rich glycoprotein	[118]
Drought	MicroRNA-835	Downregulated	*Ricinus communis*	Aquaporin	[118]
Drought	MicroRNA-4398	Downregulated	*Solanum tuberosum*	WRKY transcription factor	[118]
Salt	MicroRNA-319b	Upregulated	Switchgrass	PvPCF5	[119]
Salt	MicroRNA-390	Downregulated	Poplar	ARF3.1, ARF3.2,ARF4	[120]
Salt	MicroRNA-390a	Downregulated	Creeping bentgrass	AsTIR1, AsAFB2	[33]
Salt	MicroRNA-396c	Upregulated	Creeping bentgrass	GRF	[44]
Salt	MicroRNA-408	Upregulated	Wheat	TaCP, TaMP, TaBCP, TaFP, TaKRP, TaABP	[121]
Salt	MicroRNA-408	Upregulated	*Salvia miltiorrhiza*	NbSOD, NbPOD, NbCAT	[122]
Salt	MicroRNA-414c	Downregulated	Cotton	GhFSD1	[123]
Cold	MicroRNA-160	Downregulated	Maize		[124]
Cold	MicroRNA-319	Downregulated	Rice	PCF6/TCP21	[125]
Cold	MicroRNA-319	Downregulated	Maize		[124]
Cold	MicroRNA-408a	Upregulated	Maize		[124]
Cold	MicroRNA-528	Upregulated	Maize		[124]
Cold	MicroRNA-5125	Upregulated	Potato	ABF8011	[126]
Cold	MicroRNA-10881	Upregulated	Potato	GA3ox123158	[126]
High temperature	MicroRNA-156	Downregulated	*Arabidopsis thaliana*	SPL transcription factor	[127]
High temperature	MicroRNA-159	Downregulated	Maize	MYB transcription factor	[128]
High temperature	MicroRNA-164	Downregulated	Maize	NAC transcription factor	[128]
High temperature	MicroRNA-166	Downregulated	Maize	HD zip	[128]
High temperature	MicroRNA-169	Downregulated	Maize	SBP	[128]
High temperature	MicroRNA-172	Downregulated	Maize	AP2/ERF	[128]
High temperature	MicroRNA-396	Downregulated	Maize	GRF,	[128]
High temperature	MicroRNA-5381	Downregulated	Maize	SAC2	[128]
Heavy metals—Cd	MicroRNA-167		*Zea mays*		[129]
Heavy metals—Cd	MicroRNA-393		*Zea mays*		[129]
Heavy metals—Cu	MicroRNA-398		Grape	VvCSD1 and VvCSD2	[70]
Heavy metals—Al	MicroRNA-160		Sugarcane		[129]
Heavy metals—Al	MicroRNA-162		Sugarcane		[129]
Heavy metals—Al	MicroRNA-164		Sugarcane		[129]
Heavy metals—Al	MicroRNA-166		Sugarcane		[129]
Heavy metals—Al	MicroRNA-167		Sugarcane		[129]
Nutrients—Zn	MicroRNA-158	Upregulated	*Brassica juncea*	FUT1	[130]
Nutrients—K	MicroRNA-169		*Triticum aestivum*	Pentose pathway	[131]
Nutrients—N	MicroRNA-169	Downregulated	*Arabidopsis thaliana*	HAP2	[88]
Nutrients—B	MicroRNA-319	Upregulated	*Riticum aestivum*	MYB transcription factor	[99]
Nutrients—K	MicroRNA-319	Downregulated	*Hordeum vulgare*	TCP	[99]
Nutrients—K	MicroRNA-396	Downregulated	*Hordeum vulgare*	GRF	[90]
Nutrients—P	MicroRNA-399	Downregulated	*Arabidopsis thaliana*	Ubiquitin conjugase E2	[129]
Nutrients—Mn	MicroRNA-781	Upregulated	*Arabidopsis thaliana*	MCM2	[129]
Nutrients—Mn	MicroRNA-826	Upregulated	*Arabidopsis thaliana*	Alkenyl- and hydroxyalkyl-producing genes	[129]

## Data Availability

Not applicable.

## References

[1] Huang K., Wu T., Ma Z., Li Z., Chen H., Zhang M., Bian M., Bai H., Jiang W., Du X. (2021). Rice Transcription Factor OsWRKY55 Is Involved in the Drought Response and Regulation of Plant Growth. Int. J. Mol. Sci..

[2] Zhang H., Wu T., Li Z., Huang K., Kim N.E., Ma Z., Kwon S.W., Jiang W., Du X. (2021). OsGATA16, a GATA Transcription Factor, Confers Cold Tolerance by Repressing OsWRKY45-1 at the Seedling Stage in Rice. Rice.

[3] Tamirisa S., Vudem D.R., Khareedu V.R. (2014). Overexpression of pigeonpea stress-induced cold and drought regulatory gene (CcCDR) confers drought, salt, and cold tolerance in *Arabidopsis*. J. Exp. Bot..

[4] Laubinger S., Sachsenberg T., Zeller G., Busch W., Lohmann J.U., Rätsch G., Weigel D. (2008). Dual roles of the nuclear cap-binding complex and SERRATE in pre-mRNA splicing and microRNA processing in *Arabidopsis thaliana*. Proc. Natl. Acad. Sci. USA.

[5] Rodriguez A., Griffiths-Jones S., Ashurst J.L., Bradley A. (2004). Identification of mammalian microRNA host genes and transcription units. Genome Res..

[6] Lee R.C., Feinbaum R.L., Ambros V. (1993). The *C. elegans* heterochronic gene lin-4 encodes small RNAs with antisense complementarity to lin-14. Cell.

[7] Kim V.N., Nam J.W. (2006). Genomics of microRNA. Trends Genet..

[8] Jiang Y., Wu X., Shi M., Yu J., Guo C. (2022). The miR159-MYB33-ABI5 module regulates seed germination in *Arabidopsis*. Physiol. Plant..

[9] Betti F., Jose M., Weits D.A., Ferri G., Iacopino S., Novi G., Svezia B., Kunkowska A.B., Santaniello A., Piaggesi A. (2021). Exogenous miRNAs induce post-transcriptional gene silencing in plants. Nat. Plants.

[10] Iki T., Yoshikawa M., Meshi T., Ishikawa M. (2012). Cyclophilin 40 facilitates HSP90-mediated RISC assembly in plants. EMBO J..

[11] Park M.Y., Wu G., Gonzalez-Sulser A., Vaucheret H., Poethig R.S. (2005). Nuclear processing and export of microRNAs in *Arabidopsis*. Proc. Natl. Acad. Sci. USA.

[12] Thieme C.J., Schudoma C., May P., Walther D. (2012). Give It AGO: The Search for miRNA-Argonaute Sorting Signals in *Arabidopsis thaliana* Indicates a Relevance of Sequence Positions Other than the 5′-Position Alone. Front. Plant Sci..

[13] Bologna N.G., Iselin R., Abriata L.A., Sarazin A., Pumplin N., Jay F., Grentzinger T., Dal Peraro M., Voinnet O. (2018). Nucleo-cytosolic Shuttling of ARGONAUTE1 Prompts a Revised Model of the Plant MicroRNA Pathway. Mol. Cell.

[14] Liu Q., Wang F., Axtell M.J. (2014). Analysis of complementarity requirements for plant microRNA targeting using a *Nicotiana benthamiana* quantitative transient assay. Plant Cell.

[15] Franco-Zorrilla J.M., Valli A., Todesco M., Mateos I., Puga M.I., Rubio-Somoza I., Leyva A., Weigel D., García J.A., Paz-Ares J. (2007). Target mimicry provides a new mechanism for regulation of microRNA activity. Nat. Genet..

[16] Yan J., Gu Y., Jia X., Kang W., Pan S., Tang X., Chen X., Tang G. (2012). Effective small RNA destruction by the expression of a short tandem target mimic in *Arabidopsis*. Plant Cell.

[17] Aukerman M.J., Sakai H. (2003). Regulation of flowering time and floral organ identity by a MicroRNA and its APETALA2-like target genes. Plant Cell.

[18] Brodersen P., Sakvarelidze-Achard L., Bruun-Rasmussen M., Dunoyer P., Yamamoto Y.Y., Sieburth L., Voinnet O. (2008). Widespread translational inhibition by plant miRNAs and siRNAs. Science.

[19] Lanet E., Delannoy E., Sormani R., Floris M., Brodersen P., Crété P., Voinnet O., Robaglia C. (2009). Biochemical evidence for translational repression by *Arabidopsis* microRNAs. Plant Cell.

[20] Huang S., Ma Z., Hu L., Huang K., Zhang M., Zhang S., Jiang W., Wu T., Du X. (2021). Involvement of rice transcription factor OsERF19 in response to ABA and salt stress responses. Plant Physiol. Biochem..

[21] Ma Z., Jin Y.M., Wu T., Hu L., Zhang Y., Jiang W., Du X. (2022). OsDREB2B, an AP2/ERF transcription factor, negatively regulates plant height by conferring GA metabolism in rice. Front. Plant Sci..

[22] Singh A., Jain D., Pandey J., Yadav M., Bansal K.C., Singh I.K. (2023). Deciphering the role of miRNA in reprogramming plant responses to drought stress. Crit. Rev. Biotechnol..

[23] Arshad M., Gruber M.Y., Hannoufa A. (2018). Transcriptome analysis of microRNA156 overexpression alfalfa roots under drought stress. Sci. Rep..

[24] Feyissa B.A., Arshad M., Gruber M.Y., Kohalmi S.E., Hannoufa A. (2019). The interplay between miR156/SPL13 and DFR/WD40-1 regulate drought tolerance in alfalfa. BMC Plant Biol..

[25] López-Galiano M.J., García-Robles I., González-Hernández A.I., Camañes G., Vicedo B., Real M.D., Rausell C. (2019). Expression of miR159 Is Altered in Tomato Plants Undergoing Drought Stress. Plants.

[26] Reyes J.L., Chua N.H. (2007). ABA induction of miR159 controls transcript levels of two MYB factors during *Arabidopsis* seed germination. Plant J..

[27] Zhang M., Chen Y., Xing H., Ke W., Shi Y., Sui Z., Xu R., Gao L., Guo G., Li J. (2023). Positional cloning and characterization reveal the role of a miRNA precursor gene ZmLRT in the regulation of lateral root number and drought tolerance in maize. J. Integr. Plant Biol..

[28] Kaushal M. (2019). Microbes in Cahoots with Plants: MIST to Hit the Jackpot of Agricultural Productivity during Drought. Int. J. Mol. Sci..

[29] Romero-Romero J.L., Inostroza-Blancheteau C., Orellana D., Aquea F., Reyes-Díaz M., Gil P.M., Matte J.P., Arce-Johnson P. (2018). Stomata regulation by tissue-specific expression of the Citrus sinensis MYB61 transcription factor improves water-use efficiency in *Arabidopsis*. Plant Physiol. Biochem..

[30] Hoshika Y., Fares S., Pellegrini E., Conte A., Paoletti E. (2020). Water use strategy affects avoidance of ozone stress by stomatal closure in Mediterranean trees—A modelling analysis. Plant Cell Environ..

[31] Lertngim N., Ruangsiri M., Klinsawang S., Raksatikan P., Thunnom B., Siangliw M., Toojinda T., Siangliw J.L. (2022). Photosynthetic Plasticity and Stomata Adjustment in Chromosome Segment Substitution Lines of Rice Cultivar KDML105 under Drought Stress. Plants.

[32] Yuan W., Suo J., Shi B., Zhou C., Bai B., Bian H., Zhu M., Han N. (2019). The barley miR393 has multiple roles in regulation of seedling growth, stomatal density, and drought stress tolerance. Plant Physiol. Biochem..

[33] Zhao J., Yuan S., Zhou M., Yuan N., Li Z., Hu Q., Bethea F.G., Liu H., Li S., Luo H. (2019). Transgenic creeping bentgrass overexpressing Osa-miR393a exhibits altered plant development and improved multiple stress tolerance. Plant Biotechnol. J..

[34] Visentin I., Pagliarani C., Deva E., Caracci A., Turečková V., Novák O., Lovisolo C., Schubert A., Cardinale F. (2020). A novel strigolactone-miR156 module controls stomatal behaviour during drought recovery. Plant Cell Environ..

[35] Zhou Y., Liu W., Li X., Sun D., Xu K., Feng C., Kue Foka I.C., Ketehouli T., Gao H., Wang N. (2020). Integration of sRNA, degradome, transcriptome analysis and functional investigation reveals gma-miR398c negatively regulates drought tolerance via GmCSDs and GmCCS in transgenic *Arabidopsis* and soybean. BMC Plant Biol..

[36] Hang N., Shi T., Liu Y., Ye W., Taier G., Sun Y., Wang K., Zhang W. (2021). Overexpression of Os-microRNA408 enhances drought tolerance in perennial ryegrass. Physiol. Plant..

[37] Wang L., Bai X., Qiao Y., Si L., Yu Z., Ni C., Li T., Guo C., Xiao K. (2022). Tae-MiR9674a, a MicroRNA Member of Wheat, Confers Plant Drought and Salt Tolerance through Modulating the Stomata Movement and ROS Homeostasis. Plant Biotechnol. Rep..

[38] Waqas M., Yaning C., Iqbal H., Shareef M., ur Rehman H., Bilal H.M. (2021). Synergistic consequences of salinity and potassium deficiency in quinoa: Linking with stomatal patterning, ionic relations and oxidative metabolism. Plant Physiol. Biochem..

[39] Ma Z., Wu T., Huang K., Jin Y.M., Li Z., Chen M., Yun S., Zhang H., Yang X., Chen H. (2020). A Novel AP2/ERF Transcription Factor, OsRPH1, Negatively Regulates Plant Height in Rice. Front. Plant Sci..

[40] Pegler J.L., Oultram J.M.J., Grof C.P.L., Eamens A.L. (2020). Molecular Manipulation of the miR399/PHO2 Expression Module Alters the Salt Stress Response of *Arabidopsis thaliana*. Plants.

[41] Arif M.A., Top O., Csicsely E., Lichtenstern M., Beheshti H., Adjabi K., Frank W. (2022). DICER-LIKE1a autoregulation based on intronic microRNA processing is required for stress adaptation in *Physcomitrium* patens. Plant J..

[42] He Y., Zhou J.X., Hu Y.F., Fang C.Y., Yu Y.J., Yang J., Zhu B., Ruan Y.L., Zhu Z.J. (2021). Overexpression of sly-miR398b increased salt sensitivity likely via regulating antioxidant system and photosynthesis in tomato. Environ. Exp. Bot..

[43] Liu J.N., Ma X., Yan L., Liang Q., Fang H., Wang C., Dong Y., Chai Z., Zhou R., Bao Y. (2022). MicroRNA and Degradome Profiling Uncover Defense Response of *Fraxinus velutina* Torr. to Salt Stress. Front. Plant Sci..

[44] Yuan S., Zhao J., Li Z., Hu Q., Yuan N., Zhou M., Xia X., Noorai R., Saski C., Li S. (2019). MicroRNA396-mediated alteration in plant development and salinity stress response in creeping bentgrass. Hortic. Res..

[45] Abla M., Sun H., Li Z., Wei C., Gao F., Zhou Y., Feng J. (2019). Identification of miRNAs and Their Response to Cold Stress in Astragalus Membranaceus. Biomolecules.

[46] Wang S.T., Sun X.L., Hoshino Y., Yu Y., Jia B., Sun Z.W., Sun M.Z., Duan X.B., Zhu Y.M. (2014). MicroRNA319 positively regulates cold tolerance by targeting OsPCF6 and OsTCP21 in rice (*Oryza sativa* L.). PLoS ONE.

[47] Zhou M., Tang W. (2019). MicroRNA156 amplifies transcription factor-associated cold stress tolerance in plant cells. Mol. Genet. Genomics..

[48] Dong Y., Tang M., Huang Z., Song J., Xu J., Ahammed G.J., Yu J., Zhou Y. (2022). The miR164a-NAM3 module confers cold tolerance by inducing ethylene production in tomato. Plant J..

[49] Sun M., Shen Y., Chen Y., Wang Y., Cai X., Yang J., Jia B., Dong W., Chen X., Sun X. (2022). Osa-miR1320 targets the ERF transcription factor OsERF096 to regulate cold tolerance via JA-mediated signaling. Plant Physiol..

[50] DE Lima C.F.F., Kleine-Vehn J., De Smet I., Feraru E. (2021). Getting to the Root of Belowground High Temperature Responses in Plants. J. Exp. Bot..

[51] Cohen S.P., Leach J.E. (2020). High temperature-induced plant disease susceptibility: More than the sum of its parts. Curr. Opin. Plant Biol..

[52] Posch B.C., Kariyawasam B.C., Bramley H., Coast O., Richards R.A., Reynolds M.P., Trethowan R., Atkin O.K. (2019). Exploring high temperature responses of photosynthesis and respiration to improve heat tolerance in wheat. J. Exp. Bot..

[53] Sadok W., Lopez J.R., Smith K.P. (2021). Transpiration increases under high-temperature stress: Potential mechanisms, trade-offs and prospects for crop resilience in a warming world. Plant Cell Environ..

[54] Sadura I., Janeczko A. (2021). Brassinosteroids and the Tolerance of Cereals to Low and High Temperature Stress: Photosynthesis and the Physicochemical Properties of Cell Membranes. Int. J. Mol. Sci..

[55] Singh R.K., Prasad A., Maurya J., Prasad M. (2022). Regulation of small RNA-mediated high temperature stress responses in crop plants. Plant Cell Rep..

[56] Wang J., Xu J., Wang L., Zhou M., Nian J., Chen M., Lu X., Liu X., Wang Z., Cen J. (2023). SEMI-ROLLED LEAF 10 stabilizes catalase isozyme B to regulate leaf morphology and thermotolerance in rice (*Oryza sativa* L.). Plant Biotechnol. J..

[57] Li L., Chen G., Yuan M., Guo S., Wang Y., Sun J. (2022). CsbZIP2-miR9748-CsNPF4.4 Module Mediates High Temperature Tolerance of Cucumber through Jasmonic Acid Pathway. Front. Plant Sci..

[58] Ahmed W., Xia Y., Zhang H., Li R., Bai G., Siddique K.H.M., Guo P. (2019). Identification of conserved and novel miRNAs responsive to heat stress in flowering Chinese cabbage using high-throughput sequencing. Sci. Rep..

[59] Matthews C., Arshad M., Hannoufa A. (2019). Alfalfa response to heat stress is modulated by microRNA156. Physiol. Plant..

[60] Arshad M., Puri A., Simkovich A.J., Renaud J., Gruber M.Y., Marsolais F., Hannoufa A. (2020). Label-free quantitative proteomic analysis of alfalfa in response to microRNA156 under high temperature. BMC Genom..

[61] Arshad M., Hannoufa A. (2022). Alfalfa transcriptome profiling provides insight into miR156-mediated molecular mechanisms of heat stress tolerance. Genome.

[62] Pandey A.K., Zorić L., Sun T., Karanović D., Fang P., Borišev M., Wu X., Luković J., Xu P. (2022). The Anatomical Basis of Heavy Metal Responses in Legumes and Their Impact on Plant-Rhizosphere Interactions. Plants.

[63] Gavrilescu M. (2022). Enhancing phytoremediation of soils polluted with heavy metals. Curr. Opin. Biotechnol..

[64] Chot E., Reddy M.S. (2022). Role of Ectomycorrhizal Symbiosis Behind the Host Plants Ameliorated Tolerance Against Heavy Metal Stress. Front. Microbiol..

[65] Tighe-Neira R., Gonzalez-Villagra J., Nunes-Nesi A., Inostroza-Blancheteau C. (2022). Impact of nanoparticles and their ionic counterparts derived from heavy metals on the physiology of food crops. Plant Physiol. Biochem..

[66] Sharma A., Kapoor D., Gautam S., Landi M., Kandhol N., Araniti F., Ramakrishnan M., Satish L., Singh V.P., Sharma P. (2022). Heavy metal induced regulation of plant biology: Recent insights. Physiol. Plant..

[67] Velusamy K., Periyasamy S., Kumar P.S., Rangasamy G., Nisha Pauline J.M., Ramaraju P., Mohanasundaram S., Nguyen Vo D.V. (2022). Biosensor for heavy metals detection in wastewater: A review. Food Chem. Toxicol..

[68] Vaid N., Sudan J., Dave S., Mangla H., Pathak H. (2022). Insight into Microbes and Plants Ability for Bioremediation of Heavy Metals. Curr. Microbiol..

[69] Vega A., Delgado N., Handford M. (2022). Increasing Heavy Metal Tolerance by the Exogenous Application of Organic Acids. Int. J. Mol. Sci..

[70] Zhang L., Ding H., Jiang H., Wang H., Chen K., Duan J., Feng S., Wu G. (2020). Regulation of cadmium tolerance and accumulation by miR156 in *Arabidopsis*. Chemosphere..

[71] Kumar R.S., Sinha H., Datta T., Asif M.H., Trivedi P.K. (2023). microRNA408 and its encoded peptide regulate sulfur assimilation and arsenic stress response in *Arabidopsis*. Plant Physiol..

[72] Nie G., Liao Z., Zhong M., Zhou J., Cai J., Liu A., Wang X., Zhang X. (2021). MicroRNA-Mediated Responses to Chromium Stress Provide Insight Into Tolerance Characteristics of Miscanthus sinensis. Front. Plant Sci..

[73] Zhou M., Zheng S., Liu R., Lu L., Zhang C., Zhang L., Yant L., Wu Y. (2019). The genome-wide impact of cadmium on microRNA and mRNA expression in contrasting Cd responsive wheat genotypes. BMC Genom..

[74] Bai B., Bian H., Zeng Z., Hou N., Shi B., Wang J., Zhu M., Han N. (2017). miR393-Mediated Auxin Signaling Regulation is Involved in Root Elongation Inhibition in Response to Toxic Aluminum Stress in Barley. Plant Cell Physiol..

[75] Zinta R., Tiwari J.K., Buckseth T., Thakur K., Goutam U., Kumar D., Challam C., Bhatia N., Poonia A.K., Naik S. (2022). Root system architecture for abiotic stress tolerance in potato: Lessons from plants. Front. Plant Sci..

[76] Phour M., Sindhu S.S. (2022). Mitigating abiotic stress: Microbiome engineering for improving agricultural production and environmental sustainability. Planta.

[77] Prasad R. (2022). Cytokinin and Its Key Role to Enrich the Plant Nutrients and Growth Under Adverse Conditions—An Update. Front. Genet..

[78] Swain R., Sahoo S., Behera M., Rout G.R. (2023). Instigating prevalent abiotic stress resilience in crop by exogenous application of phytohormones and nutrient. Front. Plant Sci..

[79] Waqas M., Hawkesford M.J., Geilfus C.M. (2023). Feeding the world sustainably: Efficient nitrogen use. Trends Plant Sci..

[80] Jezek M., Allan A.C., Jones J.J., Geilfus C.M. (2023). Why do plants blush when they are hungry?. New Phytol..

[81] Johnson R., Vishwakarma K., Hossen M.S., Kumar V., Shackira A.M., Puthur J.T., Abdi G., Sarraf M., Hasanuzzaman M. (2022). Potassium in plants: Growth regulation, signaling, and environmental stress tolerance. Plant Physiol. Biochem..

[82] Lyzenga W.J., Liu Z., Olukayode T., Zhao Y., Kochian L.V., Ham B.K. (2023). Getting to the roots of N, P, and K uptake. J. Exp. Bot..

[83] Yousuf P.Y., Shabir P.A., Hakeem K.R. (2021). miRNAomic Approach to Plant Nitrogen Starvation. Int. J. Genom..

[84] Vega A., O’Brien J.A., Gutiérrez R.A. (2019). Nitrate and hormonal signaling crosstalk for plant growth and development. Curr. Opin. Plant Biol..

[85] Islam W., Tauqeer A., Waheed A., Zeng F. (2022). MicroRNA Mediated Plant Responses to Nutrient Stress. Int. J. Mol. Sci..

[86] Du Q., Wang K., Zou C., Xu C., Li W.X. (2018). The *PILNCR1*-miR399 Regulatory Module Is Important for Low Phosphate Tolerance in Maize. Plant Physiol..

[87] Hu B., Wang W., Deng K., Li H., Zhang Z., Zhang L., Chu C. (2015). MicroRNA399 is involved in multiple nutrient starvation responses in rice. Front. Plant Sci..

[88] Thornburg T.E., Liu J., Li Q., Xue H., Wang G., Li L., Fontana J.E., Davis K.E., Liu W., Zhang B. (2020). Potassium Deficiency Significantly Affected Plant Growth and Development as Well as microRNA-Mediated Mechanism in Wheat (*Triticum aestivum* L.). Front. Plant Sci..

[89] Fontana J.E., Wang G., Sun R., Xue H., Li Q., Liu J., Davis K.E., Thornburg T.E., Zhang B., Zhang Z. (2020). Impact of potassium deficiency on cotton growth, development and potential microRNA-mediated mechanism. Plant Physiol. Biochem..

[90] Ye Z., Zeng J., Long L., Ye L., Zhang G. (2021). Identification of microRNAs in response to low potassium stress in the shoots of Tibetan wild barley and cultivated. Curr. Plant Biol..

[91] Yan Y., Wang H., Hamera S., Chen X., Fang R. (2014). MiR444a has multiple functions in the rice nitrate-signaling pathway. Plant J..

[92] Prusty S., Sahoo R.K., Nayak S., Poosapati S., Swain D.M. (2022). Proteomic and Genomic Studies of Micronutrient Deficiency and Toxicity in Plants. Plants.

[93] Ninkuu V., Liu Z., Sun X. (2023). Genetic regulation of nitrogen use efficiency in *Gossypium* spp.. Plant Cell Environ..

[94] Huang S., Wang P., Yamaji N., Ma J.F. (2020). Plant Nutrition for Human Nutrition: Hints from Rice Research and Future Perspectives. Mol. Plant..

[95] Robinson R.S., Smart S.M., Cybulski J.D., McMahon K.W., Marcks B., Nowakowski C. (2023). Insights from Fossil-Bound Nitrogen Isotopes in Diatoms, Foraminifera, and Corals. Ann. Rev. Mar. Sci..

[96] Helliwell K.E. (2023). Emerging trends in nitrogen and phosphorus signalling in photosynthetic eukaryotes. Trends Plant Sci..

[97] Kong W.W., Yang Z.M. (2010). Identification of iron-deficiency responsive microRNA genes and cis-elements in *Arabidopsis*. Plant Physiol. Biochem..

[98] Valdés-López O., Yang S.S., Aparicio-Fabre R., Graham P.H., Reyes J.L., Vance C.P., Hernández G. (2010). MicroRNA expression profile in common bean (*Phaseolus vulgaris*) under nutrient deficiency stresses and manganese toxicity. New Phytol..

[99] Kayihan D.S., Kayihan C., Özden Çiftçi Y. (2019). Moderate level of toxic boron causes differential regulation of microRNAs related to jasmonate and ethylene metabolisms in *Arabidopsis thaliana*. Turk. J. Bot..

[100] Ozhuner E., Eldem V., Ipek A., Okay S., Sakcali S., Zhang B., Boke H., Unver T. (2013). Boron stress responsive microRNAs and their targets in barley. PLoS ONE.

[101] Shahid S., Kim G., Johnson N.R., Wafula E., Wang F., Coruh C., Bernal-Galeano V., Phifer T., dePamphilis C.W., Westwood J.H. (2018). MicroRNAs from the parasitic plant *Cuscuta campestris* target host messenger RNAs. Nature.

[102] Marzec M. (2022). MicroRNA: A new signal in plant-to-plant communication. Trends Plant Sci..

[103] Loreti E., Perata P. (2022). Mobile plant microRNAs allow communication within and between organisms. New Phytol..

[104] Halder K., Chaudhuri A., Abdin M.Z., Datta A. (2023). Tweaking the Small Non-Coding RNAs to Improve Desirable Traits in Plant. Int. J. Mol. Sci..

[105] Yan G., Hua Y., Jin H., Huang Q., Zhou G., Xu Y., He Y., Zhu Z. (2023). Sly-miR398 Participates in Cadmium Stress Acclimation by Regulating Antioxidant System and Cadmium Transport in Tomato (*Solanum lycopersicum*). Int. J. Mol. Sci..

[106] Giacomelli J.I., Weigel D., Chan R.L., Manavella P.A. (2012). Role of recently evolved miRNA regulation of sunflower HaWRKY6 in response to temperature damage. New Phytol..

[107] Zhang N., Yang J., Wang Z., Wen Y., Wang J., He W., Liu B., Si H., Wang D. (2014). Identification of novel and conserved microRNAs related to drought stress in potato by deep sequencing. PLoS ONE.

[108] Liu P.P., Montgomery T.A., Fahlgren N., Kasschau K.D., Nonogaki H., Carrington J.C. (2007). Repression of AUXIN RESPONSE FACTOR10 by microRNA160 is critical for seed germination and post-germination stages. Plant J..

[109] Boualem A., Laporte P., Jovanovic M., Laffont C., Plet J., Combier J.P., Niebel A., Crespi M., Frugier F. (2008). MicroRNA166 controls root and nodule development in *Medicago truncatula*. Plant J..

[110] Trindade I., Capitão C., Dalmay T., Fevereiro M.P., Santos D.M. (2010). miR398 and miR408 are up-regulated in response to water deficit in *Medicago truncatula*. Planta.

[111] Li W.X., Oono Y., Zhu J., He X.J., Wu J.M., Iida K., Lu X.Y., Cui X., Jin H., Zhu J.K. (2008). The *Arabidopsis* NFYA5 transcription factor is regulated transcriptionally and posttranscriptionally to promote drought resistance. Plant Cell.

[112] Jagadeeswaran G., Li Y.F., Sunkar R. (2014). Redox signaling mediates the expression of a sulfate-deprivation-inducible microRNA395 in *Arabidopsis*. Plant J..

[113] Barrera-Figueroa B.E., Gao L., Diop N.N., Wu Z., Ehlers J.D., Roberts P.A., Close T.J., Zhu J.K., Liu R. (2011). Identification and comparative analysis of drought-associated microRNAs in two cowpea genotypes. BMC Plant Biol..

[114] Sunkar R., Zhu J.K. (2004). Novel and stress-regulated microRNAs and other small RNAs from *Arabidopsis*. Plant Cell.

[115] Liu H.H., Tian X., Li Y.J., Wu C.A., Zheng C.C. (2008). Microarray-based analysis of stress-regulated microRNAs in *Arabidopsis thaliana*. RNA.

[116] Shamimuzzaman M., Vodkin L. (2012). Identification of soybean seed developmental stage-specific and tissue-specific miRNA targets by degradome sequencing. BMC Genom..

[117] Szabados L., Savouré A. (2010). Proline: A multifunctional amino acid. Trends Plant Sci..

[118] Ding Y., Tao Y., Zhu C. (2013). Emerging roles of microRNAs in the mediation of drought stress response in plants. J. Exp. Bot..

[119] Liu Y., Li D., Yan J., Wang K., Luo H., Zhang W. (2019). MiR319 mediated salt tolerance by ethylene. Plant Biotechnol. J..

[120] He F., Xu C., Fu X., Shen Y., Guo L., Leng M., Luo K. (2018). The MicroRNA390/TRANS-ACTING SHORT INTERFERING RNA3 Module Mediates Lateral Root Growth under Salt Stress via the Auxin Pathway. Plant Physiol..

[121] Bai Q., Wang X., Chen X., Shi G., Liu Z., Guo C., Xiao K. (2018). Wheat miRNA TaemiR408 Acts as an Essential Mediator in Plant Tolerance to Pi Deprivation and Salt Stress via Modulating Stress-Associated Physiological Processes. Front. Plant Sci..

[122] Guo X., Niu J., Cao X. (2018). Heterologous Expression of *Salvia miltiorrhiza* MicroRNA408 Enhances Tolerance to Salt Stress in *Nicotiana benthamiana*. Int. J. Mol. Sci..

[123] Wang W., Liu D., Chen D., Cheng Y., Zhang X., Song L., Hu M., Dong J., Shen F. (2019). MicroRNA414c affects salt tolerance of cotton by regulating reactive oxygen species metabolism under salinity stress. RNA Biol..

[124] Aslam M., Sugita K., Qin Y., Rahman A. (2020). Aux/IAA14 Regulates microRNA-Mediated Cold Stress Response in *Arabidopsis* Roots. Int. J. Mol. Sci..

[125] Huo C., Zhang B., Wang R. (2022). Research progress on plant noncoding RNAs in response to low-temperature stress. Plant Signal Behav..

[126] Yan C., Zhang N., Wang Q., Fu Y., Wang F., Su Y., Xue B., Zhou L., Liao H. (2021). The Effect of Low Temperature Stress on the Leaves and MicroRNA Expression of Potato Seedlings. Front. Ecol. Evol..

[127] Stief A., Altmann S., Hoffmann K., Pant B.D., Scheible W.R., Bäurle I. (2014). *Arabidopsis* miR156 Regulates Tolerance to Recurring Environmental Stress through SPL Transcription Factors. Plant Cell..

[128] Zhang M., An P., Li H., Wang X., Zhou J., Dong P., Zhao Y., Wang Q., Li C. (2019). The miRNA-Mediated Post-Transcriptional Regulation of Maize in Response to High Temperature. Int. J. Mol. Sci..

[129] Gong J., Li D., Li H., Zhou H., Xu J. (2019). Identification of manganese-responsive microRNAs in *Arabidopsis* by small RNA sequencing. Czech J. Genet. Plant Breed..

[130] Silva R.G.D., Rosa-Santos T.M., França S.C., Kottapalli P., Kottapalli K.R., Zingaretti S.M. (2019). Microtranscriptome analysis of sugarcane cultivars in response to Aluminum stress. PLoS ONE.

[131] Shi D.Q., Zhang Y., Ma J.H., Li Y.L., Xu J. (2013). Identification of zinc deficiency-responsive MicroRNAs in *Brassica juncea* Roots by Small RNA Sequencing. J. Integr. Agric..

